# Detection of Fused Genes in Eukaryotic Genomes using Gene deFuser: Analysis of the *Tetrahymena thermophila *genome

**DOI:** 10.1186/1471-2105-12-279

**Published:** 2011-07-11

**Authors:** Hannah MW Salim, Amanda M Koire, Nicholas A Stover, Andre RO Cavalcanti

**Affiliations:** 1 Department of Biology, Pomona College, Claremont, California 91711, USA; 2 Department of Biology, Bradley University, Peoria, Illinois 61625, USA; 3 Current address: Department of Environmental Science, Policy and Management, University of California, Berkeley, California 94720, USA

## Abstract

**Background:**

Fused genes are important sources of data for studies of evolution and protein function. To date no service has been made available online to aid in the large-scale identification of fused genes in sequenced genomes. We have developed a program, Gene deFuser, that analyzes uploaded protein sequence files for characteristics of gene fusion events and presents the results in a convenient web interface.

**Results:**

To test the ability of this software to detect fusions on a genome-wide scale, we analyzed the 24,725 gene models predicted for the ciliated protozoan *Tetrahymena thermophila*. Gene deFuser detected members of eight of the nine families of gene fusions known or predicted in this species and identified nineteen new families of fused genes, each containing between one and twelve members. In addition to these genuine fusions, Gene deFuser also detected a particular type of gene misannotation, in which two independent genes were predicted as a single transcript by gene annotation tools. Twenty-nine of the artifacts detected by Gene deFuser in the initial annotation have been corrected in subsequent versions, with a total of 25 annotation artifacts (about 1/3 of the total fusions identified) remaining in the most recent annotation.

**Conclusions:**

The newly identified *Tetrahymena *fusions belong to classes of genes involved in processes such as phospholipid synthesis, nuclear export, and surface antigen generation. These results highlight the potential of Gene deFuser to reveal a large number of novel fused genes in evolutionarily isolated organisms. Gene deFuser may also prove useful as an ancillary tool for detecting fusion artifacts during gene model annotation.

## Background

Fusion genes, also known as chimeric genes, are formed when the reading frames of two or more distinct genes are joined together by recombination events such as unequal crossing over, transposition, and deletion [1]. After the fusion, the new gene codes for a single, novel protein that is a hybrid of the two separate proteins, where each part performs a discrete function and has an independent evolutionary history. Although very few of these recombination events produce proteins that retain their proper function or expression pattern, on occasion the constituent genes do combine to form a new, working gene that can be passed on to offspring [2]. Generation of new multidomain proteins by gene fusions is a major mechanism by which functional complexity has evolved in multicellular eukaryotes [1,2], and many key proteins currently under research, including Hedgehog [3], Type II Topoisomerase, and RNA Polymerase [4], began as fusions of genes in the ancestors of eukaryotes.

Successful fusion requires that both halves of the new gene function properly despite the loss of expression elements from the downstream gene, which falls under control of the upstream promoter. Therefore, only fusions in which the two linked proteins can function in the same compartment of the cell, at the same developmental stage, and in response to the same stimuli will be tolerated. While it has been hypothesized that two genes with unrelated functions may merge and be retained in the genome [4,5], almost all bifunctional fusion genes seen to date show a functional relationship between the proteins that comprise the fusion. Related genes are more likely to result in a functional fusion gene, and may even confer a selective advantage to the organism in some cases. Most fused gene pairs have orthologs that are part of the same metabolic pathway, are involved in the same protein complex [6], or regulate one another's activity [5]. A selective advantage may emerge if the fused protein leads to a greater catalytic activity or more efficient co-regulation than is possible for the two independent proteins.

Given these complex requirements, gene fusions are rarely successful, and few examples exist of analogous recombinations occurring in multiple unrelated taxa by convergent evolution [2,7]. These requirements also guarantee that the split of a fusion gene into its two component proteins is much rarer than the original fusion events. Studies have estimated that gene fusion is approximately four times more common than gene fission events, in which a single gene splits into multiple, smaller coding segments [8]. The predominance of gene fusions over gene fissions is expected in part because gene fusions can result in the potentially favorable coupling of proteins with related biological functions, rather than the unfavorable separation of proteins whose shapes and functions have evolved together over time [6]. Additionally, gene fusion involves the loss of the termini of the genes being fused, a much simpler process than fission, which requires that the genes somehow obtain a promoter, terminator, start codon, and stop codon when the gene splits.

The scarce and persistent nature of gene fusions makes them ideal macromolecular markers of evolution and, like insertions, deletions, and other genomic rearrangements, they have long served as data for phylogenetic analysis. The usefulness of gene fusions in studies of this type was featured in 2003 when, following the attempts of many different research groups to locate the root of the eukaryote tree by a variety of methods, the presence of a fusion between dihydrofolate reductase (DHFR) and thymidylate synthase (TS) in plants and many protozoan species, but not in animals and fungi, supported rooting of the eukaryotic tree between these groups [9,10]. Though gene losses and horizontal gene transfer have complicated the conclusions that can be reached from these single-character analyses [11,12], gene fusions may still provide some of the most reliable information about the deepest branching taxa.

In addition to their usefulness in phylogenetic studies, gene fusions can also serve as Rosetta Stone proteins that provide information about their constituent genes. Since the fused proteins are likely to be functionally related, characterization of each constituent gene informs researchers about their homologs in other genomes [4,13]. In the majority of cases where annotation of the function of fusion proteins in eukaryotes and prokaryotes is available, the constituent proteins are involved in core metabolism, which may help researchers understand both simple and more complex biological metabolic systems [13]. In particular, fusion proteins in eukaryote genomes have been used to identify hidden protein-protein interactions [13].

Despite their important uses in evolutionary studies as powerful phylogenetic markers, and in functional studies as windows into biochemical pathways and protein interactions, few of the fusion genes present in eukaryotes have been identified and studied in depth. Researchers have previously created programs to find fusion genes in specific genomes [14,15]. However, to date no large-scale service has been made available to the public to aid in the identification of fusions in large, genome-sized data sets. Here we present a new bioinformatics tool, Gene deFuser, which we have developed for this purpose. The underlying algorithm compares BLAST results from the beginning and end of protein sequences submitted through an online interface. Putative gene fusions are displayed for the user in a convenient interface that simplifies further analysis of the candidate genes. Gene deFuser is based on programs we have used previously to identify gene fusions in the formaldehyde detoxification pathways of ciliates and diatoms [16] and in the methionine salvage pathway of *Tetrahymena *[17]. To highlight the value of this service, we present an in depth survey of the results obtained for the predicted proteome of *Tetrahymena thermophila*, which includes the identification of several new types of fusion genes.

During this survey we also identified a large number of misannotated genes models, which can be attributed to a common artifact of gene prediction software in which two genes are merged into a single transcript. Comparison of Gene deFuser results for the first and final versions of the *Tetrahymena *genome showed that about half of the artifacts found in the initial scan of the genome were corrected over time. Gene deFuser may serve as a useful tool to speed the identification of these types of artifacts in future genome projects.

## Methods

The Gene deFuser program utilizes BLAST [18] to detect similarities between the two ends of a protein and the sequences in a database of orthologous protein groups. The program compares these sequences to the KOG (eukaryotic orthologous groups) database [19], which is a subset of the COG (clusters of orthologous genes) database [20] containing groups of orthologous genes for seven eukaryotic genomes. Although newer and more complete ortholog databases exist, we chose the KOG database because it was extensively curated and the authors specifically broke down fused genes into their component KOG domains [19,20]. This allowed us to identify genes such as DHFR-TS (*DTS1 *in *Tetrahymena *[16]; see Results) that would have otherwise been masked by their presence in one or more of the species represented in the KOG database.

Gene deFuser generates a list of KOG identifiers for each end of the protein in question based on the BLAST results. The list of identifiers found for the N-terminus is then compared with those listed for the C-terminus. A typical non-fused protein will match the same KOG at both the N-terminus and the C-terminus. A protein that returns a matching KOG identifier at both ends is presumed to be non-fused and is excluded as a possible fusion gene. Proteins that do not share any KOG hit at both ends are presented in a list of candidate fusion proteins. This method obviously omits fusions that were missed during curation of the KOG database. However, any fusion genes missed due to this limitation are present in at least several of the model organisms used to generate the KOG database and, because these genomes are highly studied, these fusions are likely to have been described already. The main application of the Gene deFuser program is to identify novel fusion genes.

**Figure 1:**
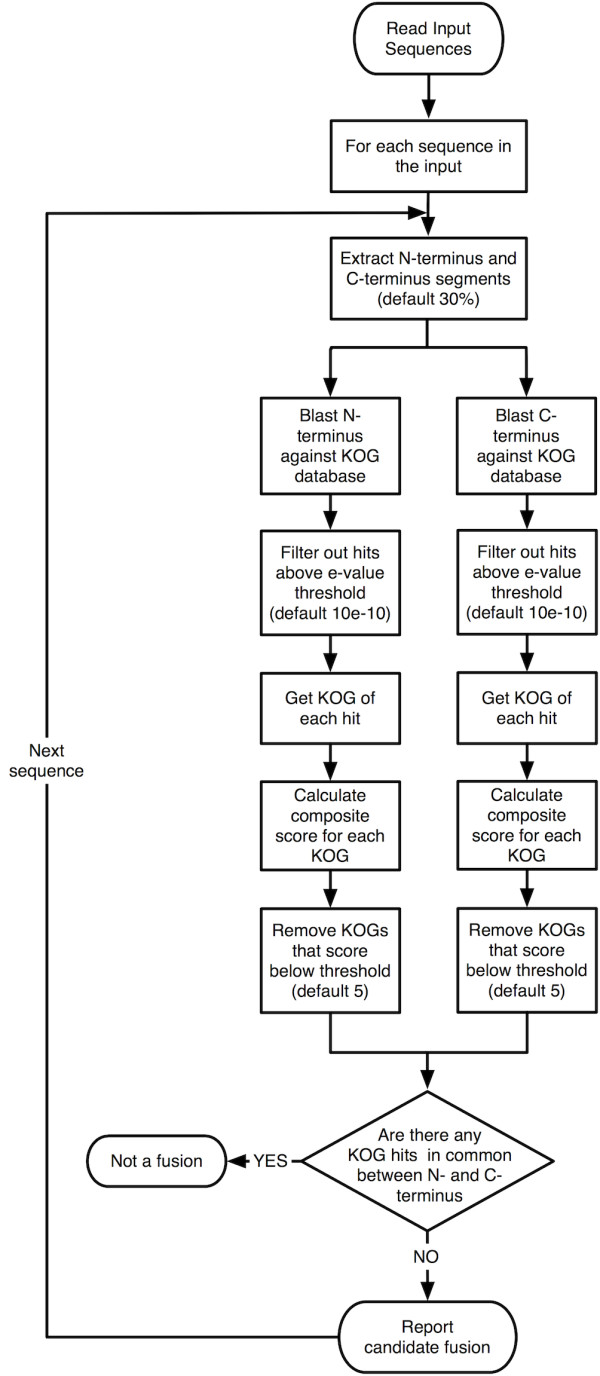
**Outline of the Gene deFuser algorithm**.

An outline of the methodology used by Gene deFuser to identify fused genes is shown in Figure 1. Gene deFuser accepts as input multiple protein sequences in FASTA format and can be used to search files that cover the size of a typical genome (~30,000 proteins). After the user submits a set of proteins, the program extracts a portion of the C-terminus and a portion of the N-terminus of each sequence to use as queries in BLAST searches. The fraction of the protein used for BLAST searches of the C- and N-terminus can be adjusted by the user, but the default is set at 30%. Using too much of the protein as a query can lead to overlap in the KOG hits on both ends and prevent the identification of fused genes; using too little of the protein can result in poor BLAST scores. This parameter must be set to less than 50% of the sequence to avoid overlap of the segments, and after experimenting with different values between 20% and 40%, we settled on using the first 30% of the protein sequence as the N-terminus query and the final 30% of the protein as the C-terminus query in our analysis of the *Tetrahymena *genome. The default value of 30% brought back 52 sequences that we believe are genuine fused genes. When we performed the search using 20%, the program only detected 19 of these 52 genes. When we increased to this parameter to 40%, the program appeared to detect a few additional fused genes; however, it missed 7 of the 52 fusions detected using 30% and returned more false positives. Based on these observations, the users are encouraged to repeat their searches using different values of this parameter.

After generating files containing the N- and C-termini of the proteins, the sequences are used to search the KOG database using BLASTP. We downloaded the KOG database on July 10, 2010 and modified the dataset by eliminating all protein sequences not assigned to a KOG. About 54% (59,838) of the 110,655 gene products analyzed to create the KOG database are included in 4,852 clusters of orthologs, while the remaining genes are not assigned to an ortholog group. The BLAST search results for the N- and C-termini that exceed a user-defined threshold (default: e-value < 1e-10) are parsed to determine the KOG of each hit, and then a combined score for each KOG is calculated using the methodology described in Zhou and Landweber [21]. Using this methodology, each KOG with a significant hit is assigned a score given by the following formula:

where N is the number of sequences belonging to the KOG group and Pi = 1-exp(Ei), where Ei is the e-value of the blast hit to a given sequence. For sequences without significant blast hits, that is, with an e-value larger than the e-value threshold, Pi = 1.

KOGs that score higher than a cutoff threshold set by the user (default = 5) are used in the second part of the analysis, which compares the KOGs that hit the N-terminus to those that hit the C-terminus of each protein. If both ends are hit by at least one KOG, and the KOGs that hit the N-terminus are different from those that hit the C-terminus, the protein is deemed a candidate fusion. We further divide the candidate fusion proteins into two categories: those that have a single KOG hit to each end and those that have multiple KOG hits to at least one end.

The program identifies all candidate fusions in the file and lists them on a web page. Each protein in this list is hyperlinked to a page that details the KOG hits at both the N-terminus and C-terminus and graphically displays the location of BLAST hits against the Uniprot and KOG databases. These results can then be examined by an expert to determine whether each candidate is a fused gene, a non-fused gene, or a sequencing or annotation error.

Because each submission can take several hours to run after data are uploaded, the user is asked to submit an email address to be notified when the job is completed. When the program finishes its run, the job number is emailed to the user for retrieval at the Gene deFuser website. Gene deFuser is freely available online at: http://DNA.pomona.edu/deFuser/deFuser.html.

To test this program and service, we uploaded and analyzed the protein set predicted by The Institute for Genomic Research (TIGR; now J. Craig Venter Institute) for *Tetrahymena thermophila *strain SB210 [22]. The current protein annotation (v.2008) [23] was downloaded from the TIGR website:

ftp://ftp.tigr.org/pub/data/Eukaryotic_Projects/t_thermophila/annotation_dbs/final_release_oct2008/tta1_oct2008_finalrelease.aa.fsa

The initial protein annotation (v.2004) [22] was also analyzed and the results were compared to the v.2008 sequences. The v.2004 sequences were downloaded from the following location:

ftp://ftp.tigr.org/pub/data/Eukaryotic_Projects/t_thermophila/Gene_Predictions/Preliminary_Gene_Predictions_Aug_2004.pep

## Results and discussion

To test Gene deFuser's ability to detect fused genes, we used it to analyze the genome of *Tetrahymena thermophila*, a ciliated protozoan evolutionarily distant from the seven eukaryotic species used to populate the KOG database. We chose *Tetrahymena *in particular because of our familiarity with the biology of the organism, its detailed genome annotation history, and our interest in several of its previously described gene fusions [16,17]. In addition to the evolutionary gene fusions we expected to find with this tool, we also attempted to identify artificial gene fusions created during the process of gene model annotation, by comparing the earliest round of gene predictions with the most recent round.

Gene deFuser detected 80 candidate fusion genes in the final annotation (v.2008) of the *Tetrahymena *genome. The raw results of these analyses can be accessed at http://dna.pomona.edu/deFuser/Results/Final_Tet/Final_Tet.html and is available as Additional file 1. Brief descriptions of the known fusions in this genome and some of the more interesting new candidate fusions detected by Gene deFuser are listed below. Prior to this analysis we were aware of nine published families of fusion genes either known or predicted to be present in *Tetrahymena *(Table 1). Gene deFuser successfully identified members of eight of these families and also revealed 19 additional families (52 new genes total) that appear to be genuine fusions. These results have been categorized and refined, and are presented in Table 1. The remaining 28 candidates either have too little similarity to sequences in the KOG or Uniprot database for us to make a valid judgment, or the architecture of the gene model (e.g. a large intron between the putatively fused domains) casts doubt on its legitimacy. It is important to keep in mind that the classification of the candidates into real fusion or false positives relies on the interpretation of available data, and that these fusions should be confirmed by experimental data if they prove to be of interest to the researcher.

**Table 1:** Fused genes detected by Gene deFuser in *Tetrahymena*

N-terminus hit	C-terminus hit	Copies found in the genome	Fusion described or predicted in *Tetrahymena*?	Gene Name	Genbank Accession
FALDH	SFGH	1	YES	*FSF1*	EAR92957
MTNB	MTND	1	YES	*MBD1*	EAS04801
dihydrofolate reductase	thymidylate synthase	1	YES	*DTS1*	EAR85731
P-type ATPase	adenylyl/guanylyl cyclase	2	YES	*PAC1*	EAS02708
				*PAC2*	EAS03660
cyclophilin peptidyl-prolyl cis-trans isomerase	SYF2 pre-mRNA splicing factor	1	YES	*CSY1*	EAR98967
SEC7-family GTPase	TBC1 domain GTPase activator	1	YES	*TBS1*	EDK31800
peroxisomal multifunctional oxidation protein	2-enoyl-CoA hydratase	1	YES	*MFE1*	EAS01180
kelch repeat containing protein	ser/thr phosphatase	2	YES	*BSU1*	EAR82584
				*BSU2*	EAS02286
fatty acyl-CoA reductase	dihydroxyacetone phosphate acyltransferase	1	NO	*ART1*	EAS00429
leishmanolysin-like peptidase	subtilisin-like proprotein convertase	12	NO	*LSF1*	EAR96678
				*LSF2*	EAR96679
				*LSF3*	EAR96681
				*LSF4*	EAR82776
				*LSF5*	EAR86010
				*LSF6*	EAR86011
				*LSF7*	EAR86012
				*LSF8*	EAR86013
				*LSF9*	EAR86016
				*LSF10*	EAR86017
				*LSF11*	EAR86018
				*LSF12*	EDK32083
ser/thr kinase	O-linked N-acetylglucosamine transferase	4	NO	*KOT1*	EAR98929
				*KOT2*	EAS07587
				*KOT3*	EAR94286
				*KOT4*	EAS05661
kinesin	ER-golgi vesicle tethering protein	2	NO	*KET1*	EAR95984
				*KET2*	EAR91273
myosin	Regulator of Chromosome Condensation (RCC1)	3	NO	*MYO11*	EAR87392
				*MYO12*	EAR93163
				*MYO3*	EAR98568
kinesin CENP-E	Regulator of Chromosome Condensation (RCC1)	2	NO	*KRC1*	EAR84240
				*KRC2*	EAR88562
calmodulin dependent protein kinase	Radial spoke protein	2	NO	*RSK1*	EAR84708
				*RSK2*	EAR84712
MAPK ser/thr kinase	Radial spoke protein	1	NO	*RSK3*	EAS01279
NIMA-related kinase	Radial spoke protein	1	NO	*RSK4*	EAR95086
guanylate-binding protein	ER-golgi vesicle tethering protein	1	NO	*GVT1*	EAR98751
ankyrin/histone H3 methyl transferase	exosome 3-5 exoribonuclease	1	NO	*AXE1*	EAR87370
ser/thr kinase	LRR-containing protein	4	NO	*LRK1*	EAR91534
				*LRK2*	EAR87255
				*LRK3*	EAR99973
				*LRK4*	EAR92811
protein phosphatase	ER-golgi vesicle tethering protein	1	NO	*LRC1*	EAR89472
PI-4-phosphate 5-kinase	tyrosine kinase	1	NO	*TKL1*	EAR94148
subtilisin-like proprotein	teneurin-1	2	NO	*CVP1*	EAR94583
				*CVP2*	EAS03363
transcription factor NF-X1	nuclear protein export factor	1	NO	*ZEF1*	EAS01176
uncharacterized protein	26S proteasome subunit	1	NO	*PLF1*	EAS02650
ser/thr kinase	Ca2+/calmodulin protein kinase	1	NO	*KFK1*	EAR81873
aarF domain containing protein	ubiquinone biosynthesis protein	1	NO	*ABC1*	EAS05302

### Known *Tetrahymena *Fusion Genes

*FSF1 *(Genbank: EAR92957) is a gene fusion that contains a formaldehyde dehydrogenase (FALDH) domain at the N-terminus of the predicted protein and an *S*-formylglutathione hydrolase (SFGH) domain at the C-terminus [16]. The initial Gene deFuser report shows that the N-terminal domain resembles alcohol dehydrogenase (ADH) Classes III and V (KOG0022 and KOG0023) while the C-terminus resembles Esterase D (KOG3101). Closer examination of these KOG hits and the list of similar proteins in the UniProt database shows that the two fused proteins function in the formaldehyde detoxification pathway. When naming the gene, we highlighted the common pathway in which these proteins function by choosing synonyms for ADH III/V (FALDH) and Esterase D (SFGH). Interestingly, this gene seems to also be fused in a distantly related group of protozoans, the diatoms, albeit in the reverse order, with the SFGH protein in the N-terminus and the FALDH protein in the C-terminus [16]. This feature shows that the two original proteins fused independently in the ciliate and diatom lineages.

*MBD1 *(Genbank: EAS04801) is a fusion of two genes in the methionine salvage pathway, methylthioribulose-1-phosphate dehydratase (mtnB) and 1,2-dihydroxy-3-keto-5-methylthiopentene dioxygenase (mtnD) [17]. This fusion seems to be unique to *Tetrahymena *and its closest relatives, as it is not present in the genome of the other fully sequenced ciliate *Paramecium tetraurelia*. Surprisingly, the *Tetrahymena *genome is lacking the enzyme that catalyses the intermediate step in the methionine salvage pathway between those of mtnB and mtnD, enolase-phosphatase E1 (mtnC). Complementation tests in yeast mutants were used to show that the fusion gene is able to catalyze the intermediate (mtnC) step of the pathway in addition to the two expected reactions, indicating a gain of function as a result of the fusion [17].

*DTS1 *(Genbank: EAR85731) is a fusion of dihydrofolate reductase and thymidylate synthase, a well-known fusion found in bikont organisms (plants, most protozoan species) but absent in unikonts (animals, fungi, and amoebas) that was used to root the eukaryotic phylogenetic tree [9]. Even though this gene is fused in *Arabidopsis thaliana*, one of the organisms used to create the KOG database, we were able to detect it thanks to the manual curation of the KOG database that broke down fused genes into their component domains.

The proteins *PAC1 *and *PAC2 *(Genbank: EAS02708 and EAS03660) each contain a P-type ATPase domain and an adenylyl/guanylyl cyclase domain. A fusion between these two genes was previously described in another *Tetrahymena *species, *T. pyriformis *[24]. In addition, the same fusion is was shown to be present in the ciliate *Paramecium tetraurelia *and in the apicomplexan *Plasmodium falciparum*, suggesting that it participates in a shared form of signal transduction in these closely related species [24].

*MFE1 *(Genbank: EAS01180) is part of a well-described peroxisomal multifunctional enzyme family with homologs in all types of unikonts, but with few homologs among the bikonts. Only the alveolates show homologs of these proteins, most likely indicating independent origins for these fusions rather than multiple losses from many paraphyletic bikonts. Functional studies have been performed on the *Toxoplasma gondii *version of the protein that demonstrate its involvement in cholesterol uptake [25].

Two copies of a serine-threonine protein phosphatase with Kelch-like repeats (PPKLs), *BSU1 *(Genbank: EAR82584) and *BSU2 *(Genbank: EAS02286), are homologs of a suppressor of brassinolide receptor kinase mutations described in *Arabidopsis *[26]. Prior to the sequencing of extensive protist and algal species, the distribution of these genes was found to be limited to plants and apicomplexans [27]. Results from Gene deFuser led us to identify *BSU1 *and *BSU2*, and further investigation led us to *BSU3 *(Genbank: EAR83784), another homolog with a variant Kelch-domain that prevented its identification by our program. Additional homologs were identified during our subsequent BLAST searches in other alveolates and in green algae.

*CSY1 *(Genbank: EAR98967) is a fusion between a peptidyl prolyl isomerase (cyclophilin) and a homolog of the yeast RNA splicing factor *SYF2*. This gene and its ortholog in *Paramecium *have been identified previously as members of a family of genes found only in alveolates, with the exception of the green algal species *Ostreococcus tauri *[28]. The specific properties of this fusion have not yet been explored, but its merit as a drug target for alveolate parasites has been noted. *TBS1 *(Genbank: EDK31800) is a small GTPase of the SEC7 family fused to a TBC1-related GTPase activating protein. Like the cyclophilin/SYF2 genes, fusions of these two secretory pathway proteins are believed to comprise a family unique to alveolates [29].

While Gene deFuser was able to identify the eight types of fusion genes listed above, it did miss two genes that we expected it to find, *TBS2 *(Genbank: EAR85277) and *CYC13 *(Genbank: EAR91121). *TBS2 *is a paralog of the *TBS1 *gene described above. Although the program did not detect *TBS2*, it did return one fusion belonging to this family. The only unique gene fusion that we expected to find but was missed by the program was *CYC13*. The *CYC13 *fusion links a cyclin protein to a cyclin-dependent kinase (CDK) and was first observed in a screen of cell cycle-specific genes in the ciliate *Eufolliculina uhligi *[30]. BLAST searches of both *TBS2 *and *CYC13 *show no similarity to known sequences for large portions of the N-terminus of each gene (32% of the *TBS2 *sequence and 35% of the *CYC13 *fusion). It is not clear whether these N-terminal sequences are indeed part of the actual proteins, but these extra sequences with no homology explain why neither protein was identified by our program. Increasing the amount of the protein sequence used to BLAST the KOG database from its default value of 30% to 45% did not help in identifying these sequences. In the case of these proteins, a sliding window approach would likely overcome this limitation in the software, as the different KOGs that hit these sequences do not overlap. Such a methodology might be implemented in a future version of the program.

### New *Tetrahymena *Fusion Genes

One of the most useful applications of Gene deFuser, and the detection of fused genes in general, is to allow the generation of hypotheses that later can be tested experimentally. For example, the fusion *MBD1 *described in the previous section was first detected during the testing of an early version of this program. Based on the lack of mtnC in the *Tetrahymena *genome, we hypothesized that this fusion of mtnB and mtnD also catalyzes the mtnC reaction. We then successfully showed this to be the case using yeast deletion strains [17]. In addition to previously described fused genes such as this, Table 1 lists several as yet uncharacterized fusions among the 80 candidate fusions detected by the program. Here we describe some of the more interesting fusions found in this list.

The first is a fusion between a long-chain fatty acyl-CoA reductase and dihydroxyacetone phosphate acyltransferase (DHPAT) (Genbank: EAS00429) that we have called *ART1*. These two enzymes catalyze sequential steps in the production of membrane phospholipids. Fusions of these genes are distributed in an odd pattern among several eukaryotic groups, suggesting either multiple evolutionary gains or losses of this fusion. The fusion is present in the ciliates *T. thermophila *(Genbank: EAS00429) and *P. tetraurelia *(Genbank: XP_001433255), but not in other alveolates whose genomes have been fully sequenced, such as the dinoflagellate *Perkinsus marinus *or the apicomplexans *Plasmodium, Toxoplasma, Babesia *or *Cryptosporidium*. The fusion is also present in the amoebozoans *Dictyostelium discoideum *(Genbank: XP_636393) and *Polysphondylium pallidum *(Genbank: EFA75040). Fusions of these genes are also found in one stramenopile, *Phytophthora infestans *(Genbank: XP_002902570), but not in other stramenopiles like *Thalassiosira pseudonana *or *Phaeodactylum tricornutum*. Likewise, a fusion is present in the excavate *Naegleria gruberi *(Genbank: XP_002683520), but not in other excavates like *Giardia intestinalis *or *Trichomonas vaginalis*.

Many of the remaining gene fusions detected in *Tetrahymena *appear to belong to expanded gene families. With twelve copies present in the genome, the most common fusion detected was a protein formed by joining leishmanolysin and a subtilisin-like proprotein convertase (Genbank: EAR96678, EAR96679, EAR96681, EAR82776, EAR86010, EAR86011, EAR86012, EAR86013, EAR86016, EAR86017, EAR86018, EDK32083), both of which are peptidases [31] that localize to the cell surface [32]. It has been noted that leishmanolysins constitute a greatly expanded protein family in *Tetrahymena *[22], suggesting that protein processing at the cell surface may be particularly complex in ciliates. It is possible that the fusions identified here might simplify these types of reactions at the cell surface. Additionally, in mice, the genes that code for both these proteins are regulated by the protein Nrf2 and are co-regulated by the anti-tumor compound curcumin [31]. These connections further suggest that these proteins contribute to a common process and that the fusion may have some significance in *Tetrahymena*.

Also present are four copies of a serine/threonine kinase fused with O-linked N-acetylglucosamine transferase (Genbank: EAR98929, EAS07587, EAR94286, EAS05661). Serine/threonine kinases phosphorylate proteins on the hydroxyl group of specific serine or threonine residues [33], while O-linked N-acetylglucosamine transferases instead attach a single β-*O*-linked *N*-acetylglucosamine to serine and threonine residues [34]. Since these enzymes could compete for the same phosphorylation/glycosylation sites, a fusion of the two catalytic domains might provide a simple way to regulate this competition.

Several of the fusions present in *Tetrahymena *involve the motor proteins myosin and kinesin. A fusion of kinesin with an ER-golgi vesicle tethering protein (Genbank: EAR95984, EAR91273) might participate in anterograde vesicle movement from the ER to the Golgi, which is known to be mediated directly by kinesin [35]. Three fusions (Genbank: EAR87392, EAR93163, EAR98568) are found between myosin and RCC1, a nuclear Ran-GEF that promotes transport of cargo across the nuclear membrane [36]. Myosins have been found in the nucleus, and some types have been shown to localize specifically at the nuclear pore complex [37]. Thus, it is possible that the myosin-RCC1 fusions identified are involved in nucleocytoplasmic transport. Two kinesin-RCC1 fusions (Genbank: EAR84240, EAR88562), on the other hand, might serve a different function. While the KOG hits in the Gene deFuser results do not specify the type of kinesin involved in the fusion, the results of the BLAST search against Uniprot show the best match is to part of Centromere Protein E (CENP-E), a kinetochore-associated kinesin. CENP-E has been implicated as a sensor that mediates the capture of microtubules at the kinetochore and relays this to the checkpoint machinery [38]. During mitosis RCC1 is responsible for the production of Ran-GTP, which is known to stimulate the release of checkpoint proteins from the kinetochores [38], thus overcoming the cell cycle checkpoint at the end of mitosis. The fusion of these proteins might provide a streamlined mechanism for cell cycle regulation during micronuclear mitosis, or may be involved somehow in the poorly understood separation of acentromeric chromosomes during amitosis.

### Detection of Annotation Artifacts

Of the 80 *Tetrahymena *genes identified by Gene deFuser, we believe that 52 are likely to represent actual fusions (Table 1). The majority of the remaining 28 (Table 2) are most likely artifacts created by faulty start/stop codon identification during gene model annotation. When viewing these genes in the genome browser at the *Tetrahymena *Genome Database [39], most show that the two domains are separated by an abnormally long non-coding region, which we believe represents intergenic regions miscalled as introns.

**Table 2:** False positives detected by Gene deFuser

Annotation Version	Number of False Positives	Accession Numbers
Final Annotation (v.2008)	28	EAR92881, EAS01798, EAR92566, EAR82879, EAS00133, EAR84691, EAR92830, EAR99356, EAR91587, EAR84275, EAR84417, EAR99401, EAR83898, EAR89871, EAR85428, EAS03452, EAS02693, EAR83154, EAR82303, EAS00607, EAR83089, EAR85121, EAR89363, EAR91270, EAR96069, EAR96106, EAR86245, EAR86074
Initial Annotation (v.2004)^1^	29 (in addition to the 28 that are still present in the Final Annotation)	EAR96923, EAR84622, EAR85248, EAR85505, EAR85282, EAR85413, EAR97343, EAR99583, EAS01392, EAS00371, EAS04594, EAR87314, EAS03022, EAS02070, EAS03869, EAR99890, EAR82527, EAR85830, EAS07404, EAR99312, EAR89578, EAR91857

^1 ^These genes were removed in the final version of the annotation. Note that even though 29 false positives were identified, only 22 accession numbers are listed. The remaining 7 false positives were eliminated before the sequences were submitted to GenBank, and thus have no accession number.

Earlier versions of the *Tetrahymena *genome are available from the TIGR (now J. Craig Venter Institute) website, and Gene deFuser analyses of these proteins return different results. The initial annotation (v.2004), contained 105 candidate gene fusions, compared with the 80 found in the current annotation (v.2008). Most of these genes (76) were present in both versions and were identified by Gene deFuser. Twenty-nine spurious fusions resulting from annotation artifacts were separated or eliminated from the annotation over the intervening period, whereas 4 new putative fusions (Genbank: EAS02650, EAS02286, EDK31800, EDK32083) were introduced into the annotation.

We believe that 25 of the 28 false positive gene fusions detected by Gene deFuser represent genes that have not yet been separated by annotators. Twenty-four of these 25 genes have an intron larger than 413 bp located between the two domains that comprise the putative fusion. The median intron length in *Tetrahymena *is 86 bp and only 8.3% of the introns in this species are larger than 400 bp (data not shown). That such large introns are located between the two domains suggests these introns were miscalled, resulting in the fusion of adjacent gene models. The presence of paired EST reads matching only the 3'end of the other gene (EAR99401) indicates that it too is an annotation artifact. One of the three remaining candidate fusions is a non-fused Ca^2+^/calmodulin dependent kinase gene present in many organisms, which Gene deFuser misclassified as a candidate fusion based on hits to several different kinase families (EAR82879). We judged the two remaining candidates to be false positives based on low BLAST scores (EAR96106 and EAR89363). Though these appear at first glance to be false positives, additional data may prove several of these 28 genes to be genuine fusions.

The detection of these annotation artifacts highlights another possible use for Gene deFuser, as a tool to aid in the refinement of gene models during genome sequencing projects. Since a large portion (51%; 54/105) of the genes detected by the program in the preliminary annotation were gene model fusion artifacts, this tool could be used following the initial annotation of new genomes to identify some of the more obvious fusion artifacts. Gene deFuser can generate a list of putative fusions for annotators to evaluate using their own criteria, which are likely to differ based on the quality of the initial annotation and the uniformity found in the lengths of introns and intergenic regions.

## Conclusions

Fused genes are a large untapped source of data for studies of molecular evolution and protein function. The new program described in this paper promises to speed the identification of fusions in a wide variety of organisms, with the most interesting results likely to come from more evolutionarily diverse species. Our application of Gene deFuser to the *Tetrahymena *genome illustrates the large number of new fusion genes waiting to be found in more exotic eukaryotic genomes. In this study alone we have identified new fusions involving a wide variety of proteins, including nucleases, proteases, motor proteins, and kinases. It is reasonable to expect an equally interesting collection of fusion genes in the genomes of other divergent eukaryotes.

## Authors' contributions

AROC and NAS conceived the study. HMWS and AROC programmed the Gene deFuser software. All authors participated in the analysis of the *Tetrahymena *genome results and helped draft and edit the manuscript. All authors approved the final version of the manuscript.

## Supplementary Material

Additional file 1 **Results of Gene deFuser for the *Tetrahymena thermophila *genome**. This zip file contains the raw results of the analysis of the *Tetrahymena *genome using Gene deFuser. To view the contents, unzip the file and open the Final_Tet.html file in the resulting folder. Click here for file
