# Supplementary material for: Detection of Fused Genes in Eukaryotic Genomes using Gene deFuser: Analysis of the Tetrahymena thermophila genome
Source: BMC Bioinformatics. 2011 Jul 11;12:279. doi: 10.1186/1471-2105-12-279 (PMC3143110; doi:10.1186/1471-2105-12-279)
Supplement: Additional file 1 — Results of Gene deFuser for the Tetrahymena thermophila genome. This zip file contains the raw results of the analysis of the Tetrahymena genome using Gene deFuser. To view the contents, unzip the file and open the Final_Tet.html file in the resulting folder. [file 1471-2105-12-279-S1.ZIP › Results/3698.m00018.html]

Gene deFuser -- Results of Job Final\_Tet

 


Gene deFuser

| Home | Retrieve Results | References | Help |
| --- | --- | --- | --- |

Back to Main Results of Job Final\_Tet

# Query Name: 3698.m00018

Candidate fusion gene

## Query Sequence:

MSHQTNQDWSKWDLIHPQVLQSLKDNNFNNPTEIQAYVLNTYRNYNDFLIASQTGSGKTLSFGIPIVSEILYDKSGAFAEKKKDQKKKEKYIRCLIIAPTRELVLQIEKHLNQISQNSKDQIRIGSIVGGISKEKQRRILSYVPDILIATPGRLWDMIDNYEHECLEKLYMLDYLVLDEADRMVELGHFDELDKILEKVYNKGEIVENKADIQRLNQLIKEKKTKTIQLRTKAELPQSSKNKLDQVGENEVMTLSGDEAKKFFASLRKNNQLDQNELKGINLDFLDDENYINSTQDKESQEGEEDEEDFEDVDDDEEEEADEEEEADEDEDDEEEEEEEEEEDDQEEQNENELENQEDEESGNEIDEEEDEENEEDQESENENQDELNEEGDHNENEDELDDQELEGEEENEQEGEEDLENLEGLNLEKPKRNTKLKVFLVSATLTKQFAGNKHKVKALSKKEKKKLNKEKKEKKKSKEEEKEEKMIPKMEALMNKVKFSGKYKVIDLTQTMLIPKNLKECKTVCVEEDKVLYLYHYLQQRPTENAIIFTNSISYAKKIVHLLEILGMKVLCMHSEMQQRQRLKKLDQFKNGQYSILVSTDVAARGLDIPSVQNVVHYQVPLDIDTYIHRSGRTARIGKAGTCYTLIGPKDGQRFQKIIKQLDKEQGIQNIEINHTERDKIRVLIDSAQQLEKSDFLVRQKQVEKAWYSKNAKLAEIEVDEEIKNELNIIKEDLSKKRVVMKQDRVEFKKIKEDIKQIHRRPNNMFLEPSQIQKYLERIEQLKASKSKNKSQQKEQNKQQQQQQHFLILASAFIYCHEHPTKEEIQNELTINLFYLKSLYSMDVSYGSGSRGQVVTATESDSEIGSYFTIKHGHGKPIQTFSKLIKYLITESQIINLQNFYKANTVKCGDIIRLEHINTGKNIYGSNHASPVSNKLEISAQGQNGESDGNDNFVIECIGQSKGSDLVGKTEFYLQHLNTSQFLTTSRRFSFNQNNCGFNCPIMNHLEVSCQRSKDNETKWKIVGINSLLINQKGIILQKSQFSNDNDKDPLNDYDDDDDDDEEHYNPSSNTKKDANVEEDEYEEVNFNRRDDL

### Significant Ortholog Group Hits and their Scores:

| N terminus | | C terminus | |
| --- | --- | --- | --- |
| [A] KOG0338 ATP-dependent RNA helicase | 36.0739175902119 | [R] KOG3358 Uncharacterized secreted protein SDF2 (Stromal cell-derived factor 2), contains MIR domains | 18.9836797491558 |
| [A] KOG0347 RNA helicase | 34.2857142857143 |
| [A] KOG0330 ATP-dependent RNA helicase | 33.3137086125519 |
| [A] KOG0348 ATP-dependent RNA helicase | 32.353582264499 |
| [A] KOG0333 U5 snRNP-like RNA helicase subunit | 31.3153281631221 |
| [A] KOG0340 ATP-dependent RNA helicase | 29.6046268134043 |
| [A] KOG0331 ATP-dependent RNA helicase | 29.3094624990926 |
| [A] KOG0341 DEAD-box protein abstrakt | 28.8514583168628 |
| [A] KOG0343 RNA Helicase | 28.8290810680077 |
| [A] KOG0337 ATP-dependent RNA helicase | 27.2845108756275 |
| [A] KOG0346 RNA helicase | 25.9950245994213 |
| [A] KOG0335 ATP-dependent RNA helicase | 25.6321299779824 |
| [A] KOG0342 ATP-dependent RNA helicase pitchoune | 23.4203651233207 |
| [A] KOG0345 ATP-dependent RNA helicase | 20.6220040206651 |
| [A] KOG0334 RNA helicase | 20.1182031371522 |
| [A] KOG0336 ATP-dependent RNA helicase | 19.6040612036049 |
| [A] KOG0339 ATP-dependent RNA helicase | 17.4632801189632 |
| [A] KOG0344 ATP-dependent RNA helicase | 12.4391098963484 |
| [A] KOG0350 DEAD-box ATP-dependent RNA helicase | 11.7536696726451 |
| [K] KOG4284 DEAD box protein | 9.21034774596445 |
| [J] KOG0327 Translation initiation factor 4F, helicase subunit (eIF-4A) and related helicases | 5.63167478238594 |

#### Graphs (click to enlarge):

|  |  |
| --- | --- |
| BLAST of Query Sequence | Location of Ortholog Group Hits |
|  |  |

Contact: Andre Cavalcanti\_\_\_\_\_Last Modified September 14, 2010
