# Supplementary material for: Detection of Fused Genes in Eukaryotic Genomes using Gene deFuser: Analysis of the Tetrahymena thermophila genome
Source: BMC Bioinformatics. 2011 Jul 11;12:279. doi: 10.1186/1471-2105-12-279 (PMC3143110; doi:10.1186/1471-2105-12-279)
Supplement: Additional file 1 — Results of Gene deFuser for the Tetrahymena thermophila genome. This zip file contains the raw results of the analysis of the Tetrahymena genome using Gene deFuser. To view the contents, unzip the file and open the Final_Tet.html file in the resulting folder. [file 1471-2105-12-279-S1.ZIP › Results/3730.m00064.html]

Gene deFuser -- Results of Job Final\_Tet

 


Gene deFuser

| Home | Retrieve Results | References | Help |
| --- | --- | --- | --- |

Back to Main Results of Job Final\_Tet

# Query Name: 3730.m00064

Candidate fusion gene

## Query Sequence:

MIKFQSQIRGSFDEESRLLLIMNKEFIIYFNNYMNSSQTEEEAILQETLEFLQEELEQNKGFKITNKYGGRGGFGLVLFVEEMSDPSKKYAIKAQSIINTMTGKVNKNLLKQCEDEATILRSCDHKNIVQIHSDFVIGLYHFIQMNLCQCSLQDWIDQNTQPINDMLFLHYVNQIIDGIEYLHNKDYVLRDLSVRNILITTDNIVKLCDFGLAKKYDDLLKSKVLYTQTARGVFLYFPPELQEDLNAQKSQIKQTKKGDIWAFGICLMILGGIKYNQLLNLQNNNYQVSDAPFLSESSNQLIKFILNKDPQKRPSFPEIREKIFSLYYGRNGMLNESLGSKSTQSSSGSELIDVLQSSKLKFYSESQTILTQITEANQVLQSPINQETNRKLSSSLEQFPISNKKDDSEKIPEVQLHKKAFSVQVAIDKNIDIDFMETNIIFNKYSKHLKEYPNSVQALLTLGYLNAYAFSNYELSKKYFEEAIQIDGNEIDAYLGICTCIILNRELKYISKAKSYLNKCLSIKKQYWRTYYIYAWLLILANKEKEALNYLIQGLQIENQSVELLSLQLIQYRHQNQINTLDLAEKVANLNKKHNPVVFQRLGSFYSNEMQNYTKAIYFYKQAYEINKKDLLTHIHLADNYYKNKDSWEAEQYLINAQILYPENSILLNFLGQKEVDLANQEQLFKKSISLDPYNKWALYNLARCQLSLGNFESAIKYYKKVLEQDPEDEQANAGIGLVLGFYLQDFNQAIEHYKIAINTNFLKLEYLINLANLHLNIKDLDSAQSYINQCMQINSNTPKIYEILCKIEQQKGNSLKAVEYIQKQMELEPQNADVYHRLGQLYHQSNPEEAKKNYIKSLQLDPKQKMVNYRLGLLEKEFTQQIKYYQNELIINPQNIEAISAVAMSLQCQGKYDLALQFLQKGLKRDPNNYILYKNIANVYSIQRKYYESIESYKQALNLNAQNIELLFLLANTYFLSGQTENAIDNYKEAIKLNPSYHQSYFELGKIYEELKQYQQAVEQFQVYLQYQPNSSETYYKIGMIEYLHFKNIQKAQICFIQSIQLNPNNNSSCYRYLGLIQNELGDYKQAKQNFLQAIEINKNEEDLYFILAQISYNYFKDIWQAIEYLEKYLQLFPNQEKQEQLLNEWYLKVNNTVRARETYEKQIQENPQNISAIMKIASIEYQVKNYHKSIFQYNKVLEIDPNNKLSLYNIGLCFKQLEKYEKAVEYFQNVIKIYQSFSLAYYQIGEIEQRYLNNPKKAIKFLRKAIELKGQDADSYFYLGLAFDKIDYKYEALECFQKLLDCSPKYPTADFCKKYISENIIVKKKKYKETTLEENDCKVF

### Significant Ortholog Group Hits and their Scores:

| N terminus | | C terminus | |
| --- | --- | --- | --- |
| [T] KOG0576 Mitogen-activated protein kinase kinase kinase kinase (MAP4K), germinal center kinase family | 14.9770090139724 | [GOT] KOG4626 O-linked N-acetylglucosamine transferase OGT | 69.1162346213924 |
| [T] KOG0574 STE20-like serine/threonine kinase MST | 10.9490172265041 | [R] KOG2003 TPR repeat-containing protein | 6.35844849717323 |
| [T] KOG4278 Protein tyrosine kinase | 6.6330984854143 | [R] KOG1124 FOG: TPR repeat | 5.79035013880508 |
| [T] KOG4257 Focal adhesion tyrosine kinase FAK, contains FERM domain | 6.35844849717323 |
| [R] KOG0597 Serine-threonine protein kinase FUSED | 5.52620864757867 |

#### Graphs (click to enlarge):

|  |  |
| --- | --- |
| BLAST of Query Sequence | Location of Ortholog Group Hits |
|  |  |

Contact: Andre Cavalcanti\_\_\_\_\_Last Modified September 14, 2010
