# Supplementary material for: Detection of Fused Genes in Eukaryotic Genomes using Gene deFuser: Analysis of the Tetrahymena thermophila genome
Source: BMC Bioinformatics. 2011 Jul 11;12:279. doi: 10.1186/1471-2105-12-279 (PMC3143110; doi:10.1186/1471-2105-12-279)
Supplement: Additional file 1 — Results of Gene deFuser for the Tetrahymena thermophila genome. This zip file contains the raw results of the analysis of the Tetrahymena genome using Gene deFuser. To view the contents, unzip the file and open the Final_Tet.html file in the resulting folder. [file 1471-2105-12-279-S1.ZIP › Results/3717.m00027.html]

Gene deFuser -- Results of Job Final\_Tet

 


Gene deFuser

| Home | Retrieve Results | References | Help |
| --- | --- | --- | --- |

Back to Main Results of Job Final\_Tet

# Query Name: 3717.m00027

Candidate fusion gene

## Query Sequence:

MDLFKKIFQLQNDEESKSQTNKQGARHGQGMMHYNNGDKYEGEWYNNLRQGQGKQYYYNGDFYTGSWNKDKKEGHGQYFYSNNERYEGDWSNDLRHGKGKYFYRNGDIYDGSWVKGKKEGKGRMTYYNNEEYDGDWLNDKKHIKNSTQNNESKEENNHKRRKNGQEDNSNTNSYEEKNSIDQIRKVISNGSERKNSSPKNKKSIQDDKDYEYDYNKPSSNNIKEEIQNNLFSNNQINSNILESQQLQYTNQYHSNRDNDFAIATNVEESVSKSNIIATEVNDTYRTNGQSLQISIDDNKAIDILDIMESYEILNNFPQDLREWNTGHICKWLDSIGLGEFSPSFESNQICGQSICMLTEQELEQSIGMKALGKRKIFLKYQEMLKKYYNKKLSKDIKQFIHKHQSRFKKGEKSLQFLKQNQQNYRSIREEDESISSHKNSLNSNQINQMENFMLLEEDEDEDHSGGKKQKDKDKKNNSYRNSDDEQNKEKNENNKKNQKIKKKKRDKGSNNSSSNDESDYSQSDNQKVSGLRSRENSGSNLVNVKEDQKEKKQKRNSPQYQYINNNNNNFEENYKYKVELQKDKSKNFAGDEDEEIHKFLKFQKNKSQTEQQKHHNNHISKVQSDSTYNSHKLKIQQINNVYCEDKLFQDIKVEKKQNHKQSGDYAQNIQSESLLTEQNQELAYTISNGNYQNQDIQTAQDLNQNNIQEEEIKNQKQLSKEVNKTQSLDNSYVKKKKKDDKLGPRDGKFNNNSIESIDTLEQKQHKSDDAASVNQMNSPDIRQNDLIKSLQPDFINTKPFILNFNQSINQNENTLFKLGIQKDLFISDKNDSQKTGLTKITSSNQSGGQTLINQSSGAIGNPTAQNSSVENNSGQNKGMQISNNQLSSSSSSSDESSDNEPNKQVVNKRSKGDGGASISSQDSDTNSKLKQRQTPQQKFKKENTKDGSRNPSKDKATPQIKHKDKDKDKMKEVSKEKSRLRREEKQQQLDQLLRDMGINKKLIINYQELDFGKKIGEGSYGQVFKGTWAKTQVAIKQFGKQNSKFHLRKVQDFISEVRVINNLRHPNIVLYMGVCFYQSQYFMITEYLQEGSLYDHLHIKHTAFSEAKQIDMIEDMALGMVYLHGRKVMHCDLKSSNVLIDENWNVKLCDFGLSRIKSTLNKKKNARKNEGLIGTPQWMAPEIMRREQYQEHSDVYSFGMIMWEIATRKVPYLGLSHQQIYGTVGYDENYQVEIPVRGIPRYLNLMKKCLRRNPQERPTFQEVVEEIFKIKKDLKDNNKKIIRFFD

### Significant Ortholog Group Hits and their Scores:

| N terminus | | C terminus | |
| --- | --- | --- | --- |
| [T] KOG0229 Phosphatidylinositol-4-phosphate 5-kinase | 49.9595153702233 | [T] KOG4278 Protein tyrosine kinase | 40 |
| [R] KOG0231 Junctional membrane complex protein Junctophilin and related MORN repeat proteins | 42.3205768944019 | [T] KOG4721 Serine/threonine protein kinase, contains leucine zipper domain | 40 |
|  |  | [T] KOG0574 STE20-like serine/threonine kinase MST | 35.5555555555556 |
|  |  | [T] KOG0197 Tyrosine kinases | 27.7777777777778 |
|  |  | [T] KOG0196 Tyrosine kinase, EPH (ephrin) receptor family | 26 |
|  |  | [T] KOG0192 Tyrosine kinase specific for activated (GTP-bound) p21cdc42Hs | 23.3707865168539 |
|  |  | [T] KOG0577 Serine/threonine protein kinase | 20 |
|  |  | [T] KOG0193 Serine/threonine protein kinase RAF | 20 |
|  |  | [T] KOG0579 Ste20-like serine/threonine protein kinase | 20 |
|  |  | [T] KOG0201 Serine/threonine protein kinase | 12.6315789473684 |
|  |  | [T] KOG0576 Mitogen-activated protein kinase kinase kinase kinase (MAP4K), germinal center kinase family | 10 |
|  |  | [TU] KOG1026 Nerve growth factor receptor TRKA and related tyrosine kinases | 10 |
|  |  | [R] KOG0597 Serine-threonine protein kinase FUSED | 8 |
|  |  | [T] KOG0194 Protein tyrosine kinase | 7.82608695652174 |
|  |  | [T] KOG0200 Fibroblast/platelet-derived growth factor receptor and related receptor tyrosine kinases | 7.55555555555556 |
|  |  | [T] KOG0578 p21-activated serine/threonine protein kinase | 7.27272727272727 |
|  |  | [T] KOG0198 MEKK and related serine/threonine protein kinases | 6.77966101694915 |
|  |  | [T] KOG1025 Epidermal growth factor receptor EGFR and related tyrosine kinases | 5.71428571428571 |
|  |  | [T] KOG1095 Protein tyrosine kinase | 5.33333333333333 |

#### Graphs (click to enlarge):

|  |  |
| --- | --- |
| BLAST of Query Sequence | Location of Ortholog Group Hits |
|  |  |

Contact: Andre Cavalcanti\_\_\_\_\_Last Modified September 14, 2010
