# Supplementary material for: Detection of Fused Genes in Eukaryotic Genomes using Gene deFuser: Analysis of the Tetrahymena thermophila genome
Source: BMC Bioinformatics. 2011 Jul 11;12:279. doi: 10.1186/1471-2105-12-279 (PMC3143110; doi:10.1186/1471-2105-12-279)
Supplement: Additional file 1 — Results of Gene deFuser for the Tetrahymena thermophila genome. This zip file contains the raw results of the analysis of the Tetrahymena genome using Gene deFuser. To view the contents, unzip the file and open the Final_Tet.html file in the resulting folder. [file 1471-2105-12-279-S1.ZIP › Results/188.m00073.html]

Gene deFuser -- Results of Job Final\_Tet

 


Gene deFuser

| Home | Retrieve Results | References | Help |
| --- | --- | --- | --- |

Back to Main Results of Job Final\_Tet

# Query Name: 188.m00073

Candidate fusion gene

## Query Sequence:

MSNKKKAIQVYLDFMVGSKPLGRVVFELFTDLTPKTAENFRGLCTGDYGQSGLSGRNAKLWYENSKIHRIVDNFCIQGGDITNGDGTGGFSIYGRHFADEDLSRRHTCAGLLSMANSGRNTNSSQFFITLKAAPHLDGKHVVFGQVIDGMDIVRQIAKVPVDLNDRPKIPVIIRQCGEVGDKRAFLRDDPFSKQSYEEIQQRKTQAEELRRQGIDPEEYFKKLDEKEEKLNEIDESLKVLQQLEEKINEVKEKPITEMNESTIKEDVIASDKSKLNEKQKDRLREIRLKLNEAKKLNSLAVLEEEKRAVDPYYEKNKKKEIIKDKAQKKQEELIKKGFEKNQQYMEQSAIKQEIFQGKEKKNTTDNFGWNVFNSDTLYGAYEKRCKNLVFNEEQYKQQVENPHQEFQPTEESLHRLQQDMDQQQFFNINLDFQKKLIFLKQNRKAKKKEFSRRRGFDEQNIDFIDERNRVFNNKIKRHFSEYTKEIKANLERGTAL

### Significant Ortholog Group Hits and their Scores:

| N terminus | | C terminus | |
| --- | --- | --- | --- |
| [O] KOG0879 U-snRNP-associated cyclophilin type peptidyl-prolyl cis-trans isomerase | 40 | [DA] KOG2609 Cyclin D-interacting protein GCIP | 15.8466290286877 |
| [O] KOG0881 Cyclophilin type peptidyl-prolyl cis-trans isomerase | 39.2087306778234 |
| [O] KOG0546 HSP90 co-chaperone CPR7/Cyclophilin | 36.6666666666667 |
| [O] KOG0880 Peptidyl-prolyl cis-trans isomerase | 33.8461538461538 |
| [O] KOG0883 Cyclophilin type, U box-containing peptidyl-prolyl cis-trans isomerase | 29.846827436561 |
| [O] KOG0884 Similar to cyclophilin-type peptidyl-prolyl cis-trans isomerase | 29.3376635683944 |
| [O] KOG0111 Cyclophilin-type peptidyl-prolyl cis-trans isomerase | 24 |
| [O] KOG0865 Cyclophilin type peptidyl-prolyl cis-trans isomerase | 21.7776214599951 |
| [O] KOG0882 Cyclophilin-related peptidyl-prolyl cis-trans isomerase | 17.3262226539922 |
| [O] KOG0885 Peptidyl-prolyl cis-trans isomerase | 13.8748104777713 |

#### Graphs (click to enlarge):

|  |  |
| --- | --- |
| BLAST of Query Sequence | Location of Ortholog Group Hits |
|  |  |

Contact: Andre Cavalcanti\_\_\_\_\_Last Modified September 14, 2010
