# Supplementary material for: Detection of Fused Genes in Eukaryotic Genomes using Gene deFuser: Analysis of the Tetrahymena thermophila genome
Source: BMC Bioinformatics. 2011 Jul 11;12:279. doi: 10.1186/1471-2105-12-279 (PMC3143110; doi:10.1186/1471-2105-12-279)
Supplement: Additional file 1 — Results of Gene deFuser for the Tetrahymena thermophila genome. This zip file contains the raw results of the analysis of the Tetrahymena genome using Gene deFuser. To view the contents, unzip the file and open the Final_Tet.html file in the resulting folder. [file 1471-2105-12-279-S1.ZIP › Results/151.m00103.html]

Gene deFuser -- Results of Job Final\_Tet

 


Gene deFuser

| Home | Retrieve Results | References | Help |
| --- | --- | --- | --- |

Back to Main Results of Job Final\_Tet

# Query Name: 151.m00103

Candidate fusion gene

## Query Sequence:

MHQYQQQQDLDLQKVKQELEDQKQLRLLSQGKGTLGQIFIAEHIIQKSYHAVKIFSLQDEKNQISAKKLDNAKREAELLKNVNHPNIIRYVDEFQINLNYFIVTEKCVKNLEQFIQEFQSQLASISRHQLVNFACQILSAINYLHQKNYIMRELSIRSILIDSVNQIKLCDFGQAKQVQEEISYSLILRNIPKSVIFYYPPELVKQVEANVEQNKKEFMLYFNKDIWAFGIWLYLLAGATQKNCYKLMKKGYQALQCLDQSLSQILSQTLSLDPFKRPSILKLVDFFQSFRKQLWNSEQAKIVYCQFKKMRKQNQLYLAYQLISICFQMQPKNEKYSFQLANVQDQLKYQEAINQYLKCIELNPKEDSYLLFLGIAQRNQGMFDEAIKSFKECLNLNPNYDVCYFNLGIVYKIKRIYDEAIKQFQKCLRLNRKYDACLFNLGISYKKKGMLNKAIKQYKKCLSLNPKYDACHYNLGIAYKKKGMVDEALKSFQDCIDLNPKYGACYYNIGNIYLMKDLLEEAIAQYQKCLTLDPNYEACFFNLGVIYKKKCMIEEAVNLFEKCLSLNQKYYACYYNLGLIQNEKGIIDEAIKLFLKCLDINPNFDACYYSLGVAYKNKGMLNDAIKQFQNCINLNSKLDYCFFELGNVQYDQGMLDESVQSYLKCIDLNQSFQNCSLKLGNIYQQKGMLDEAIKQFQKYLSIDSENDTCQMNLGICLEKTGKLDEAIKQFQNCLDLNPKNEICYLKIGDVYRKKAMINEAISAYKKCLEINPKNDICCLSLGICLENSNKINEAIECYLNCIEINPQNDICYMNLGNLYQNQNELDKAIESYYKCLNVNPQLDSCYYYLGEAQYKKSLFDESIKSYLKCLEINPNNEACYLSLGQTYQNQGMINEAILIYEKSLNLNIKIDVCCLNLGVCYEIKGRIDEAIKKYQQSIEINPANDVCFLNLGNAYLNKGMFDEAIQAYQKCLQLNPKKEACYLNLGNVYQIKGELDKAIKCYQKCIILNPKKDICYLNLGNAYQNKGNLEESIKNYQKCLNLNPKNDTCLENLGNAFKNKGMIEEAIKQYRFCLQLNPNKYSCYLNLGNTYQKKGMLDEAIECYNKCININPNNETSYANLGLCYLSKDMKYDAIKQFQKCLQINPNNKTCLISLQKMQNKKN

### Significant Ortholog Group Hits and their Scores:

| N terminus | | C terminus | |
| --- | --- | --- | --- |
| [T] KOG4278 Protein tyrosine kinase | 23.1403983013795 | [GOT] KOG4626 O-linked N-acetylglucosamine transferase OGT | 248.174956217853 |
| [R] KOG0597 Serine-threonine protein kinase FUSED | 19.7239549472794 | [DO] KOG1155 Anaphase-promoting complex (APC), Cdc23 subunit | 40.8980702771203 |
| [UT] KOG0662 Cyclin-dependent kinase CDK5 | 18.1338300686877 | [R] KOG1124 FOG: TPR repeat | 29.5071738071232 |
| [T] KOG0616 cAMP-dependent protein kinase catalytic subunit (PKA) | 15.2929635009609 | [D] KOG1126 DNA-binding cell division cycle control protein | 26.8538164078009 |
| [D] KOG0591 NIMA (never in mitosis)-related G2-specific serine/threonine protein kinase | 14.0466780544941 | [R] KOG1129 TPR repeat-containing protein | 18.1288479078797 |
| [R] KOG0611 Predicted serine/threonine protein kinase | 13.7141590429778 | [R] KOG1125 TPR repeat-containing protein | 12.9902110697039 |
| [T] KOG0579 Ste20-like serine/threonine protein kinase | 12.517272229059 | [DO] KOG1173 Anaphase-promoting complex (APC), Cdc16 subunit | 12.8868404067137 |
| [R] KOG0586 Serine/threonine protein kinase | 12.3639899005975 | [O] KOG0548 Molecular co-chaperone STI1 | 9.69453754623515 |
| [D] KOG0580 Serine/threonine protein kinase | 9.76169587977902 | [A] KOG1127 TPR repeat-containing protein | 9.01647539988968 |
| [T] KOG4236 Serine/threonine protein kinase PKC mu/PKD and related proteins | 8.48000627273461 |
| [D] KOG0575 Polo-like serine/threonine protein kinase | 8.09918306997713 |
| [T] KOG0197 Tyrosine kinases | 7.0602140094312 |
| [R] KOG0593 Predicted protein kinase KKIAMRE | 6.76586214491199 |
| [R] KOG0589 Serine/threonine protein kinase | 6.27715915455654 |
| [T] KOG0576 Mitogen-activated protein kinase kinase kinase kinase (MAP4K), germinal center kinase family | 6.22267469899189 |
| [T] KOG1095 Protein tyrosine kinase | 5.3353789727236 |

#### Graphs (click to enlarge):

|  |  |
| --- | --- |
| BLAST of Query Sequence | Location of Ortholog Group Hits |
|  |  |

Contact: Andre Cavalcanti\_\_\_\_\_Last Modified September 14, 2010
