# Supplementary material for: Detection of Fused Genes in Eukaryotic Genomes using Gene deFuser: Analysis of the Tetrahymena thermophila genome
Source: BMC Bioinformatics. 2011 Jul 11;12:279. doi: 10.1186/1471-2105-12-279 (PMC3143110; doi:10.1186/1471-2105-12-279)
Supplement: Additional file 1 — Results of Gene deFuser for the Tetrahymena thermophila genome. This zip file contains the raw results of the analysis of the Tetrahymena genome using Gene deFuser. To view the contents, unzip the file and open the Final_Tet.html file in the resulting folder. [file 1471-2105-12-279-S1.ZIP › Results/3813.m01443.html]

Gene deFuser -- Results of Job Final\_Tet

 


Gene deFuser

| Home | Retrieve Results | References | Help |
| --- | --- | --- | --- |

Back to Main Results of Job Final\_Tet

# Query Name: 3813.m01443

Candidate fusion gene

## Query Sequence:

MNQKQDFVQQQIQEKDKQMNALDKFENTQIQQQPDQISLFLFSHEQIALNLPGHPASNYSIMDIINFQTDFSFYEKIFDRKNRKILQAPVARMYGNDKQGNSVCLHIHGVNQISHLIQNDKTFLDKFIDLIEECYYTLYSVSEKQKKFLMSTTPIILNYEIIYKKDIYGYHSTEEPFLKIYFYNPKMIKKLVGILESGVVMNIEFVIYEGHLTHFLKLYSDLDIKGLREIKLLKYSLRKNENQALQQLTKEREICKYYLNSKNKFNSMTKVSTCDVEIDVYYKNILNYIFLQEEKSEENFIEMCSDLELAKNCKQRTRECSVQINKSLEMFWKEEKQMQEDLFGIKSMKATQTIDYTDNIESLFMNEDDEKEFNLSRFQFNGFSGQSTFLQEQKQKKLQEKQELLKKFNNDIYWINQLSLNKNINKQFRQHIQEFIDQSKQQLDKPMNAFLNQEILKNFHKVQKQKKDEGIHSIINRNKQYGKRKVKYYEKLLKGQVKELDLLPEDQQEDSENDQQNEDIDQYDSDSDEQSSSSDDDNDENINDEDDNNNFQEKDSKNMEIKISPNYYQVFLEKLVEEKIEKLNITKKRTLNEFLQGNSEKLKHEEDQKVKKHIKLQYEKQTNQNNIQIWRDNFNAVKNNMEVEDTAFSECQYCKQIKYLCRCNLNESTKQDIQIVQQFFEKPQNQAKRFVLKYKPKCPSFNQIMATGISKQVEHQYAYYENLEDLFEHYNLKNKSVQRNLYFAQILEKDIIKTPQHKQYYVGIQNNLKLPKYFRAYITEDYDSQQQFQETKQRFRYQKKCPSFKQISADIIKSNKKQNKQLLNVTPVTKNWLSPFSHSQKQEKLTENQQNQNKKLTQYHEDITMYTFEIFAKSTPDMLPDPSSDPINFIVFDLENFKRTQGIILVDEINCHEPIDKVRKYYGFNDSMFKTIIVTKNEEELLLTFIKQIHIFNVDIITGWDIEKKSLFYFSHRCWQLGIDVMDILSRCPKNSFNILRAFKLADCLIEEQFQNDSAKGYSQQSMSFDSNQSFKLQNVAMQSIKQSHRSAKQTGFFANFKLNFEIAGRMIINTWRLVKHDYKLFCYELESVVNYVFQERIPIFSNWSLTQMYNHEDIKQRVFVFEYMMKRLMKTKDIIDHIQIINTCAEETKLYGFDFQSAICKGHQFRIEHIMNRVTDLLGYKLMSASRYGVQTQRMLECSPLTLEPPKLFYVDPVIVLDFISLYPSIMIAYNLCFSTCLGYIDDNFQKGGFKKLGVQKQWDVQFEELLKKHNYDIDELMKDIFVAPNKVAFVKKSVREGVLSQITHEFFFTRQFIKGNMGKYKQNPLYNHIYNKLQQRQKSLKLFMCVIFGYTGATFSGRMPLGDLADSIIQIGKFLLNQAINLINNSKKWGAQVIYGDTDSVFVNIKGVSVNDAIKIGKEIESEVSKMFPYPLKLKYEKTYQNLVILTKKRYAGFFVENETDEPKFEAKGLEVMRKDGCQALSIIMKNCLETLLKNKNLSAIKLYLNGEWQKLLNDQFSYRDLIISKECKLENYTMPPPHARIALKEMKRDPQTKPKYGQRIKYFIINNPQSQRLYDCVVSVNEFMKNYRHQINLNEYLEKQINSALGRLFSTFDVDIQDWVQRIKKSEIKSHNILAIQNKQQKGGLSSEFQKQKKQFQLNHYYLKEICIACSNKTKNQICENCLQDPSALIFILTQKKKINEMKLQKLIDKCTNCCNLSQIEEMPQCIQYECYTYFEKKNAEEVVEVINPYLEQYTSIWYSLKKADQNFQTIQIKDQNNLQENTESYQYLVDKGRLYLKQGKLEEAQNLFQLALKYYPKTDYLSHHLLGFTFYQQGKFQDALQKFNESLQINPLQVDIYNTIGSIYDQQNMKDQAIKQYQKALEIQPSYYTALLNLGNLYFWDKNMVKEANECFQKALDINPNSLQVLKRAALFYYSNNQFQEAIQNYEKALSIDPQDYEIFGCLAQVYHQIGNIQKAIKILEKAIKQNPRNHQFHYDLGNYSSEVGLKNEAIQCYLNALEINPEFYQALNNLGGEYIFMERLEEAQSCFLKILETYPQDFNALIQLVVLCIERGMIEEAKDYLQKCLLNNNLDYDACNGIAQCYEALGMIEEAIFWCEKALKINPNSVDVLSNIALLHFMNGNTEESKICFEKTLKIKPDHSYALTNLGFIYYLQGDYSKAISFYQQSIEIDPSMHHGFNNLGLIYQHQGLAEQAKQQYEKALQILPNFAQALNNLGSIYYKNGKIEDAIEYYKKAQQVDPQFLEPYKSLGYIYQKIGMVVEAKNMLDQLTQLKQNQQKIFDFKNYISITQL

### Significant Ortholog Group Hits and their Scores:

| N terminus | | C terminus | |
| --- | --- | --- | --- |
| [L] KOG0968 DNA polymerase zeta, catalytic subunit | 14.1210222382283 | [GOT] KOG4626 O-linked N-acetylglucosamine transferase OGT | 184 |
|  |  | [P] KOG2002 TPR-containing nuclear phosphoprotein that regulates K(+) uptake | 29.4823624895436 |
|  |  | [R] KOG2003 TPR repeat-containing protein | 28.8839001313419 |
|  |  | [R] KOG1124 FOG: TPR repeat | 19.6369738738538 |
|  |  | [DO] KOG1155 Anaphase-promoting complex (APC), Cdc23 subunit | 11.9142496917087 |
|  |  | [A] KOG1127 TPR repeat-containing protein | 11.2718405396489 |
|  |  | [D] KOG1126 DNA-binding cell division cycle control protein | 10.6142124112194 |
|  |  | [R] KOG1129 TPR repeat-containing protein | 10.2835655127423 |
|  |  | [V] KOG0624 dsRNA-activated protein kinase inhibitor P58, contains TPR and DnaJ domains | 6.15882218990855 |

#### Graphs (click to enlarge):

|  |  |
| --- | --- |
| BLAST of Query Sequence | Location of Ortholog Group Hits |
|  |  |

Contact: Andre Cavalcanti\_\_\_\_\_Last Modified September 14, 2010
