# Supplementary material for: Detection of Fused Genes in Eukaryotic Genomes using Gene deFuser: Analysis of the Tetrahymena thermophila genome
Source: BMC Bioinformatics. 2011 Jul 11;12:279. doi: 10.1186/1471-2105-12-279 (PMC3143110; doi:10.1186/1471-2105-12-279)
Supplement: Additional file 1 — Results of Gene deFuser for the Tetrahymena thermophila genome. This zip file contains the raw results of the analysis of the Tetrahymena genome using Gene deFuser. To view the contents, unzip the file and open the Final_Tet.html file in the resulting folder. [file 1471-2105-12-279-S1.ZIP › Results/3714.m00089.html]

Gene deFuser -- Results of Job Final\_Tet

 


Gene deFuser

| Home | Retrieve Results | References | Help |
| --- | --- | --- | --- |

Back to Main Results of Job Final\_Tet

# Query Name: 3714.m00089

Candidate fusion gene

## Query Sequence:

MTIINKDILIKIIQHLNCLPTCDSTCSFDALGNYCLGSSQNCNACQSQYAKIDASITTQFSIYATCNQNKNAFGYYFNQANNILYQCNPQCYRCVDGGTCESCQQNYVYIQSTKSCVSQCPTGQYPDENRLCQQCPQGCSRCINYFICVQCQQSTYYLQYFYNSTSQQGQYNCTTTLTNQFPQQDNLYPISDSQPNQVSIFEQCDPQCNGCQNASQNCIACQPNYSSNQSNQATFFCVQSCNDGFYYQQSNTQKQCQPCSNKCVSCTTNPGCDRCSSDTYKQLNAIGANECVNVCQDGYYADQILLECLKCDQSCATCFGPSSYNCLTCSTNYYKAGSYQCIQPKCGDGILTQETEQCDDGNNLNNDGCSSTCQIEPGWKCCIANDPDPTKWICSFYDQSQGNYNQLLNLIYYKSKCLTTCGDGFRVGPQQGGTEQCDVGPSPTPTAGCDSNCQIENGYSCIGGSPTTQDVCTYLCSKQCITCQTNYLSNSQLTGTCIQCPQGMYAYQEQCLQKCPNNYFNDSTSGLGFCVQSCPSNSFINGQNCVSNLYFINYVQELFQNKQIAQCDPGQLINNNMCFDKCDQGTYSSKDPITSQITCSICHFSCLACSDGGPNFCTSCQIGYKQIDLGNNQLKCVLQCKNGYKQDPQNSNKCIVCMLNCDKCISPYYQYNDDCVQACPDGTFQNEETKTCDKFQNPKVSFIYDPPLQGSNEIGINQDYKITAVTSSVYPIVSMTWSIENQSPTFLSGLQLNTNTQSTMGITTSFINLETFSKPVISINLLVNGKQQFIINHLSDTLTIQTKYWPTPQQISYQIQSIGQSTGKLLNIDSGSSTTDLSVKYIFPFLKASETYTIQLYLWNQNIQLNTTESLTVTTNSVPIRPLDQIFGKAFNLNQLTDYQVNTLISTIIAQYQQQLQQNNQFLNNLELNIYYYQKFSIPIKCRQQQLCAVGTCNDSTQNSLSCDCQTGYSGRFCSFTTDQFNQIQSLGTILNSQLQSMAFSEQQYIQIALNTTMLTDIGQNIFTNVGNSLSQYINKITPDSLINNYQSYIQIIGQIAEEQLVLSGSNSQQTQLFNSVYQSFQTLNNKRINALNQNQPFLSVNYQQNLYCYLVTFQVANITSPTKLIASSQWNLATQNKSYKIQLINSSTRRSLQVIQDGNKIFNLTGIIQNDVIETEVTIPKSIIKASSVQQVQLQIHRWQMNPRQSQASNMQGNLTNIETHFIEVQSNLNQKYINKNDNTTYILYMLPRIRNQIDDQTILLIENPFSCFSFDTTQNIWTNSTCVYIQQTYYHIYCSCPTINTVFFATRNFDLFQPLQSESLIALIQKLVSNSGLWMVIFVLLSFLMCWLGTKDSNDPDYIDKKTFRRLTPLLSVFFVRYPLKPVRFRIIQAFLTIFTQMLIESLLYLVYEDSFTIDAVPNYALYGVLAAIPFNYLVGLFSIWAQYSESEENIMLEIQNEKMEGTLSLYTYHIVSNQQEADAIKKLYQNKQDNKEIKISSAAKYSKYKQINKEASINKEPEAFSSIQNNTNHLHSNSSLNKSEQESKSIGQQPKILIMQPDLIKSRVITFIAVCILFLVAEAAGVLLLIQVNSNSSATGYFIGSVFVGFVIDFIFFDFSIALAAKYIKPIFKLITYRGIWFKMPKDIRMKKDYLAIQNENMLDKSSISSRFQNSQHHNINKSGNNHNQEAESENNNKNLVSNTKNINQLLNDRDNEDLDVEEINKNQLDQFKDHSEESMNYFCGSTRDSSNIERLSQKEQFYSGISNNQQQNQTQQNQLNGYNMSFYQQDQGQKKERSQSEIVVRNFDTALSYEYQMSQKDKTDEAYPNKMANRSSSASSNSFLFNQHGHKYSFNSDFISDEKYFENQVKRSSNNQALTISNNQSQVLLIQTESNIKDFEIKNIKEKILEENNSDNNSSYVHKYKEPNYSSAKKQIEQLNEHVRTLEKQNQLLQSTNRNQSSTSLDVKNYQLESHSNFISPNSKQDRFVNDILYENKQLSISIKQLEDKIQNYRYIDIKYNQLQTKVVLLANQLEQTSQFLEQRGFESQELKQKYFKLEQDYINLQKTLYSLKGQNTQGRSIQDVFEEQVKQYQSVEKQNLQYKIQIRELEELSLKQQEQLTQLQQKYNNQEQTQQINLKFLKDENSSLKVKLQEEQQQKELLRNIQDNKQVPLLKQQAQQMIDQITKLSDQLKVKDQDLIKAKNNLLEIQYSQQNENIQLKQKLQESELNLQYWKTKCADTEAEKNMWISQYNQSVLNQIAQDKKIQDSKIEIDNQHKEYLKVQIDEQKKEVDSLKILNSNLQLENKILSEQINQLKESVTSFQYKSQFKDQYLGSLIRDLEQLNQIVRQENQEKLSKSNIEEQLNLEQGRNEILRKHCQELELKIKTTQNNLGSNQSIESTEDTEKVKRNKKQKQNKQVELDNNSNTVRSNSKNEEIQKYIQQQDQINNLEKQILQLQIYESQDQQNQIRIKELNEKINQLQLQNLELEDKIKLKSKVDTMLSANQNQNQEDEKKMIDLLVQIEELNKIINSQNSKEQQLIEENSLLKSQLKTLQDLQIQNEELVKFQKNYIKEKQIIEDRLKDLDQVIEKNNSQYKNKIEEQRITIKNLEKRIDLFKSQSVNNIKDKTNFEQDFYSIQNINAEYSQQVQKLKNELEVLQVKYKQLQVDNSQLKVQSEQQNKVYSQNKKEFGELQFQLNQANSKVLLLENQIKQKIVADNDNEKQSDTIKGNDYFSSELIKLQAEFKNQDKKLEIEKNQNLILQNNIINLQKIINQKQQELKNEKEESNTKFNLLEQNVNGLIQQNQSLKQQISEKTQSLQVLQNNYDQATKKLKEKMQVQELYEKQQNQLNQLIQDSACLQQELSRSKEEFNKKVQEQIDLESQIFNYKEQIFKLELQLKKYQSNLNVDNISSASNTLQDQNDQRSQDLLNQIQRINSQNMQLKQSLDLVIEQNNKLADEVLLQKNQMNSMNQQILQYQQTISQLEDEQNEKTKKSKKIDMHSNNLELQIRELEDQIRSKDLLVNSIQLELDNYRITSSQKLSSIQNQVDEKTKKVEKLEQIYQESEKNFQKHLVELSKKDQAIKNLQIQMSALESKNLHVVNDLNLIQQREATLNTQVKQLVQENSQLRAQKIISDASQMNLSALIKENQILKEQHLTKIALIKYQVKNLFNHELKQSIEKVKQISLQKEQQLHATIQILQQQLQEEKSKYESLQTTYTSRNKKSQEHINADELKQNQIIISNKYQSTDLILDPQQIFSTLSQVVNDNTSLKKFIEEMSVKILQMQETLHNSLELLWKEQNVFMSCNLDTQEEQTNSSIQHHQIEKQVKQIIQNLNQLKMHKDEQNVSSGNLNYQDSSSLPISLQAINKQQEDDGNNQSCLLKQITQFTQYTQQELENVVQQIEFLEKQNKLLKNQLQNTQKENNKLLIELKVVKEQYINTKYSTILHSSIMKQEGEQSQELKEQNQNILQQLSNTNISEEQKIVYLMMQNKQLIEMIKTFQINEAKRSEQLNSSNIQLYMALKDIANFKSKNFNFETKIPFFESEQSII

### Significant Ortholog Group Hits and their Scores:

| N terminus | | C terminus | |
| --- | --- | --- | --- |
| [O] KOG3525 Subtilisin-like proprotein convertase | 28.3346727433295 | [S] KOG4674 Uncharacterized conserved coiled-coil protein | 44.2803992275108 |
|  |  | [U] KOG0946 ER-Golgi vesicle-tethering protein p115 | 22.3978955015322 |
|  |  | [S] KOG4643 Uncharacterized coiled-coil protein | 15.0254725314486 |
|  |  | [Z] KOG0161 Myosin class II heavy chain | 13.7405740994104 |
|  |  | [ZR] KOG4568 Cytoskeleton-associated protein and related proteins | 12.923835845487 |
|  |  | [KRT] KOG4572 Predicted DNA-binding transcription factor, interacts with stathmin | 8.44281198006471 |
|  |  | [T] KOG0612 Rho-associated, coiled-coil containing protein kinase | 8.28406597104322 |
|  |  | [U] KOG4809 Rab6 GTPase-interacting protein involved in endosome-to-TGN transport | 7.88596092969672 |

#### Graphs (click to enlarge):

|  |  |
| --- | --- |
| BLAST of Query Sequence | Location of Ortholog Group Hits |
|  |  |

Contact: Andre Cavalcanti\_\_\_\_\_Last Modified September 14, 2010
